# The Medical Community’s Role in Communication Strategies during Health Crises—Perspective from European Union of Medical Specialists (UEMS)

**DOI:** 10.3390/idr15040037

**Published:** 2023-07-03

**Authors:** Ilia Nadareishvili, Theodore Bazas, Nicola Petrosillo, Vojko Berce, John Firth, Armando Mansilha, Mihaela Leventer, Alessandra Renieri, Mauro Zampolini, Vassilios Papalois

**Affiliations:** 1 Georgian Association of Medical Specialties, David Tvildiani Medical University, 0159 Tbilisi, Georgia; 2 Panhellenic Medical Association, Committee of Health and Welfare, Municipality of Filothei Psychiko, 15452 Athens, Greece; 3 Fondazione Policlinico, Universitario Campus Bio-Medico, 00128 Rome, Italy; 4 Division of Pediatrics, University Medical Centre Maribor, 2000 Maribor, Slovenia; vojko.berce@guest.arnes.si; 5 Department of Cellular Pathology, Royal Free Hospital London, London NW3 2QG, UK; johnfirth@nhs.net; 6 Faculty of Medicine, University of Porto, 4200-319 Porto, Portugal; vascular.mansilha@gmail.com; 7 UEMS Section of Dermatology, Dr. Leventer Centre, 011216 Bucharest, Romania; mihaelaleventer@drleventercentre.com; 8 Department of Medical Biotechnologies, University of Siena, 53100 Siena, Italy; renieri@unisi.it; 9 Physical and Rehabilitation Medicine Section of UEMS, Department of Rehabilitation, Hospital of Foligno, 06034 Foligno, Italy; mzampolini@gmail.com; 10 Imperial College Renal and Transplant Centre, Hammersmith Hospital, Imperial College Healthcare NHS Trust, London W12 OHS, UK; vassilios.papalois@nhs.net; 11 European Union of Medical Specialists, 1040 Brussels, Belgium

**Keywords:** COVID-19, UEMS, communication, infodemics

## Abstract

The COVID-19 pandemic was complicated by the spread of false information leading to what became widely called an “infodemic”. The present opinion paper was written by an ad hoc international team united under the European Union of Medical Specialists (UEMS) umbrella and reflects the organizations’ effort to contribute to the resolution of these issues, by highlighting and reflecting on them and by suggesting the medical community’s necessary activities resulting in the formulation of effective future communication strategies. The importance of physicians’ and other health workers’ role and mission as educators and leaders in communities in critical situations should be reassessed and upgraded. We need to equip future doctors with strong and sustainable leadership and communication skills through relevant undergraduate and postgraduate education programs, in order that compliance with preventive medical advice is increased. To avoid possible politically and otherwise biased communication in health crises of the future, European nations should establish independent advisory bodies providing evidence-based advice and participate in communication campaigns. Medical and other health professional organizations should build organizational and personal capacities of their members to enable them to reliably inform and adequately educate governments, populations, civic society, employers’ and employees’ organizations, schools and universities, and other stakeholders.

## 1. Introduction

Communication, the promotion of health literacy and health education incorporated in all levels of education, is at the core of reaching public trust and compliance with advice/directives from governing or health authorities. Communication (and specifically risk communication—“the exchange of information among interested parties about the nature, magnitude, significance, or control of a risk”) is one of the core pillars in public health emergency response and received significant attention as one of the weakest points during the COVID-19 pandemic as communication effectiveness determined outcomes of COVID-19 pandemic response [1,2,3,4].

The way how, during the COVID-19 pandemic, health-risk and crisis management was communicated, in regard to communication content and approach, has a decisive impact on how messages are received, perceived, and acted on by the public [5]. Considering Europe’s ethnic, cultural, political, language, religious and other diversities, communication approaches cannot be fully standardized and generalized, and efforts are required to address these specific differences and needs. We should note that people’s attention to COVID-19-related news and information was reduced with time, because people became oversaturated with information, then “desensitized”, and frustrated by perceived ostensible ineffectiveness of certain preventive measures and a few contradictory medical opinions. European Union (EU) institutions have limited influence on member states’ health systems, and the EU has no binding power regarding health. The EU struggled to harmonize its COVID-19 response among the member states as trust and transparency were missing [6].

## 2. Mass Media, Social Networks, and Other Online Resources and the COVID-19 “Infodemic”

Traditional mainstream mass media, such as TV, radio, and the printed press, have been utilized throughout Europe to continually inform the public about COVID-19, the evolving pandemic, and related laws and regulations. Often mass media invited health specialists of various backgrounds, caliber, qualifications, and experience to comment, e.g., on COVID-19-related regulations and make predictions about the course or the end of the pandemic. Overall, traditional media work did both good but also bad to prevent disease spread and promote health. Nevertheless, as regards pandemic prevention, mass media are not a substitute for education, but a complement to it.

Legacy mass media were also heavily complemented or in some settings alternated with increasingly influential online resources, social media, and networks [7,8,9,10,11]. Intensified use of social media during the pandemic could be explained by increasing isolation and emotional changes [12]. Social media allows easy and rapid/immediate spread of information, expression of opinions and feelings, etc. It is thus an easy way to spread “fake news”, spread misleading information, manipulate people’s opinions, and push certain agendas and propaganda narratives, including about COVID-19 and the response measures. A large portion of the content spread through social media has no useful information in it, and worse, much of this content spreads misleading or false information with the potential for harm. Most rumors, conspiracy theories, and “fake news” are spread through social media, mostly by individuals themselves, rather than in an organized manner. Associations between use of social media as primary source of information and believing in various conspiracies as well as not following recommendations guided by research-evidence received significant research attention. Despite somewhat varying findings, it should be safe to say that increased social media use was associated with negative outcomes and reluctance to follow disease related public health recommendations and mandates [11,13,14]. The “World Health Organization named too much information including false or misleading information in digital and physical environments during a disease outbreak” an “infodemic” and suggested the following 4 types of infodemic management activities: listening to community concerns and questions, promoting understanding of risk and health expert advice, building resilience to misinformation, and engaging and empowering communities to take positive action [15]. The phenomenon of social media celebrities and “influencers”—people with high number of subscribers to their blogs/vlogs and with a powerful outreach of their content—allows people of different backgrounds to influence processes in societies. At the same time, the pandemic management was affected by increasing activity of “bots”, i.e., autonomous programs on the internet that can interact with other systems or users, and by growing numbers of “bot posts” [16]. There is little control on what information is spread, whom it reaches, and what is the eventual impact on people’s behaviors, health, and lives [17]. It is pointed out that the Strengthened Code of Practice on Disinformation of 2022, which empowers industry to adhere to self-regulatory standards to combat disinformation, is considered by the European Commission to fulfill its expectations set out in its relevant guidance and to contribute to the fight against misinformation, disinformation, and information influence operations [18].

Governments, international organizations, and other stakeholders involved in pandemic management and policy development recognized social media’s power and used social media to inform populations. As the official messages and information required periodic revision, the removal or modification of government or international authority published information was sometimes used as a reason to spread conspiracies while “to some degree future expert messages were discredited”. Even though academic and public health organizations, as well as physician unions, are not absent from social media, their content and publications has been regularly receiving less spread and outreach compared to content posted/published by persons not relevant to medicine or public health [19,20].

COVID-19 quickly became a good stage for “public physicians” and self-proclaimed “public health experts” who attained particular attention in this era [17]. Many of these people managed to enlarge their audiences in a short time and, in some cases, attain celebrity status. Many of them not only prematurely spread novel still untested and uncorroborated medical research findings but also confused policy processes and spread of misinformation, contradictions, and public concerns by employing sophistry. Part of the high impact they had could be accounted for by pre-existing mistrust of politicians and governments, in certain countries.

Trust and credibility of many health specialist speakers (some of whom have used efficient front line communication practices, in some settings on behalf of governments due to higher levels of trust from societies compared to trust to governments and politicians) suffered [5].

Pandemic policy and medical advice communication were complicated by extreme dynamism and complexity of occurring events. Many messages became obsolete in a short time, because often information was soon changed as the pandemic evolved. Sometimes they had not been based on adequate real knowledge or scientific data or had not been explained to the public by phrasing them in common terms.

## 3. What Can the Medical Community Do?

In any health crisis, it is very important that information spread by governments, doctors, and other responsible authorities is accurate, evidence-based, and reliable and is communicated in a clear, substantiated, compassionate, and transparent way. The following six Crisis and Emergency Risk Communication framework principles were suggested in 2021 (adapted from the United States Center for Disease Control Crisis and Emergency Risk Communication Manual): Be first; Be right; Be credible; Express empathy; Promote action; Show respect. Communication should be timely, accurate, and disseminated effectively, aiming to minimize the gap between knowledge and action [3]. Transparency and clear language are both important in any critical situations as cooperation depends on trust as we are addressing people of many different ethnic, social, education, cultural, and economic backgrounds. Attention and efforts should also be turned to swift correction of misinformation and timely information of people. All the communicated content and approaches should be adapted considering differences of the target communities and subpopulations. Uncertainties should be acknowledged [3,5,19]. Relevant stakeholders’ engagement in message development and communication should be considered and pursued. This is not only important in order to increase people’s trust and adherence to advice but also to address the “fake news”, rumors, infodemics, and other misinformation [18]. Conspiracy theories can be responded to, directly, by distributing true information and explaining the false content of them. Doctors, public health officials, and governments should “acknowledge powerful social, psychological, and political forces that undermine compliance” and plan long-term interventions that also work to leave as little “empty information space” as possible in order not to leave room for biases conspiracy elaboration and widespread [21].

The importance of physicians’ and other health workers’ role and mission as educators and leaders in communities in critical situations should be reassessed and promoted. It is also important to equip future doctors with strong and sustainable leadership and communication skills. Physicians should take a more proactive role as local community leaders and educators. Primary care and occupational health doctors might have a particular role in communication with working people, patients, and their families [22].

## 4. Way Forward as Seen by the Ad Hoc Working Group of the European Union of Medical Specialists (UEMS)

The present opinion paper was written by an international team of medical specialists united under the European Union of Medical Specialists (Union Européenne des Médecins Spécialistes—UEMS) umbrella. UEMS is a non-governmental professional organization representing national associations of medical specialists in the European Union and in associated countries. This paper reflects the organizations’ effort to contribute to the global response to the COVID-19 pandemic as well as future pandemics and health crises. The manuscript is a result of the work of one of the ad hoc COVID-19 sub-working-groups, which worked solely on a voluntary basis.

We recognize that the COVID-19 pandemic is not an exclusive event, and we must become ready for the future global health crises related to communicable diseases (keeping in mind the ongoing ones). Further public health crises (e.g., military conflicts, displacement of people, and emerging and re-emerging communicable and widespread non-communicable diseases) will continue to affect our nations and societies. In this context, UEMS believes that to avoid possible politically and otherwise biased communication strategies, our nations would benefit from the formation of independent advisory bodies, which would provide evidence-based advice and participate in communication campaigns and activities, monitor and assess their effectiveness, and revise them as appropriately. Measurable, objective criteria for the selection of members of experts’ advisory committees should be established and formalized in each European country. Such committees should include medical specialists, specialists in epidemiology, occupational medicine, and public health, and non-medical specialists, e.g., in social psychology, bioethics, health economists, communication, health risk management, risk communication, education, and civil protection.

It is understandable that individual physicians in most specialties need to expand their capacities to address the spread of misleading information and enhance health literacy of people. All practicing physicians, at all consultations with any patient or at all preventive medical examinations, need to be able to adequately explain valid medical evidence (in a way suited to each individual to overcome denialism and non-compliance of any origin) including the necessity not to harm others—despite any personal or political beliefs. Medical and other health professional organizations, national, regional, and international, have a crucial role in the process of bringing together available time, material, and human resources and bring the voices and messages of health workers to those addressed: governments, populations, civic society, employers’ and employees’ organizations, schools and universities, and other stakeholders. These functions are in the core of missions of the majority of such professional organizations. Therefore, UEMS will continue relevant work and efforts, including through coordination, harmonization, and unification of the work of national member associations. A communication and dissemination network could be formed to be proactively activated, depending on the intensity of the situation.

There is a need for introducing relevant training modules in medical and journalism schools in order to avoid the, often unintentional, spread of misleading and false information. We recommend the development of a code of ethics and guidelines pertaining to presentation of news and interviews at the time of health crises, such as a pandemic, which could be developed in collaboration with the medical community in all European countries, with the European Federation of Journalists and national journalists’ associations.

We further suggest that all pandemic data pertaining to communication issues should be centralized in a handbook of communication principles and good practices and subsequently broken down by legislation, countries’ profiles, and communication channels. In addition, starting from this material, we recommend that a necessary action plan be built up, to be used in a crisis, in each European country, in all public and private enterprises and organizations, in national health services, pertaining also to all types of societal activities, including inter-country transportation.

We call on European and international organizations, including the World Health Organization, the International Labour Organization, and UNESCO, to invest in regional and global coordination efforts to address disinformation. We call on academic institutions, education management, and accreditation institutions to strengthen communication between physicians and patients and between societies at large. Leadership, soft skills (i.e., conflict management, public speaking, and team building), global health, information technologies in medicine, and other relevant content of teaching, learning, and assessment in undergraduate and postgraduate medical curricula including Continuing Medical Education/Continuing Professional Development courses need closer attention and possibly enhancement. Cooperation with civic society and community organizations, including student- and youth-led ones, should be considered and utilized when revising and enhancing medical education programs.

We also call on public mass media and social media companies to prioritize people’s health and lives rather than benefits given by resonating news and publications. Information and sources of information spread through media need to be carefully screened for potential bias or unprofessional actions from real or “fake” and unprofessional medical specialists, relevant laws, and regulations.

All the stakeholders should work for a long-term enhancement of health literacy and education in populations, improve communication, perception, and critical reasoning skills in people, and ensure regular communication among the health workers, community leaderships, governments, and the populations at large.

## 5. Concluding Remarks

The global community, including Europe, faced the COVID-19 pandemic largely unprepared, despite the fact that we knew that it was a matter of time when the pandemic would happen and despite assertions of preparations. The medical community and politicians should accept that a crisis of COVID-19 scale might happen again, at an unknown point in time in the not too distant future. Hence, the medical community should start spearheading the creation and the introduction of pandemic prevention strategies, utilizing also appropriate communication opportunities with mass media, politicians, and all stakeholders. As we were writing these lines in May 2023, the world was returning to the “old normal”, i.e., to our previous normal lifestyle. Nevertheless, the question remains if instead of reaching out for the “old normal” we should transform into the “new normal” of our life and professional activities. Moreover, the future of our nations and societies will heavily rely on physicians’ and other health workers’ stance and interventions, unity, and collaboration to appropriately: inform decision makers and influence health and welfare policy as well as dully educated societies to protect health, lives, and future generations. Professional organizations and unions should build capacities and enhance leadership roles to guide educational policy and other interventions leading to better readiness and performance in health crises of the future.

## Data Availability

Not applicable.
